# Associations between dietary patterns and sarcopenia in aging populations: a community study from eastern China’s Huzhou city

**DOI:** 10.3389/fnut.2025.1677335

**Published:** 2025-11-25

**Authors:** Qi Zhang, Meihua Yu, Zheng Huang, Yimei Shen, Xinfeng Zhu, Jingyi Yun, Jingying Ding, Rong Chen, Lijie Shi, Lingyan Wang

**Affiliations:** 1 Huzhou Center for Disease Control and Prevention, Huzhou, Zhejiang, China; 2 Changxing Country Center for Disease Control and Prevention, Huzhou, Zhejiang, China; 3 Deqing Country Center for Disease Control and Prevention, Huzhou, Zhejiang, China; 4 Anji Country Center for Disease Control and Prevention, Huzhou, Zhejiang, China

**Keywords:** sarcopenia, dietary patterns, aging populations, principal component analysis, restricted cubic spline plots

## Abstract

**Background:**

As the global population ages, sarcopenia has become a significant health issue. Although diet is a key factor, evidence linking specific dietary patterns to sarcopenia risk in older adults is inconsistent, especially in unique regional diets like China’s. The study aimed to identify main dietary patterns and explore their associations, including possible dose–response relationships, with sarcopenia risk among older adults in Huzhou, China.

**Methods:**

In 2024, a convenience sample study in Huzhou collected fasting blood samples and administered food frequency questionnaires (FFQs). Principal component analysis (PCA) identified dietary patterns, which were divided into tertiles. Logistic regression (LR) and restricted cubic spline (RCS) models analyzed the link between these patterns and sarcopenia.

**Results:**

Our study involving 1,030 participants aged 60 and above, sarcopenia prevalence was found to be 21.2%. PCA identified four distinct dietary patterns: Pattern 1 (plant-based whole grain-legume), Pattern 2 (traditional high meat-egg), Pattern 3 (vegetable-freshwater aquatic), and Pattern 4 (high dairy-low refined grain). Adjusted LR analysis demonstrated that greater adherence to Dietary Patterns 2 [T3 vs. T1: adjusted odds ratio (aOR) = 0.63, 95% confidence interval (CI): 0.40–0.98, *p* < 0.05] and 3 (T3 vs. T1: aOR = 0.48, 95% CI: 0.29–0.78, *p* < 0.01) was inversely associated with the occurrence of sarcopenia. Conversely, moderate adherence to Dietary Pattern 4 (T2 vs. T1: aOR = 1.73, 95% CI: 1.10–2.72, *p* < 0.05) showed a positive association with sarcopenia. No statistically significant association was identified for Dietary Pattern 1. RCS analysis revealed linear dose–response relationships for Dietary Pattern 3, which correlated with a decreased risk of sarcopenia, and for Dietary Pattern 4, which correlated with an increased risk (both *p* for overall association < 0.05; *p* for non-linearity > 0.05). Additionally, significant linear or non-linear associations were observed between Dietary Patterns 2–4 and sarcopenia diagnostic indicators, including physical performance, muscle mass, and grip strength.

**Conclusion:**

Dietary Patterns 2 and 3 were negatively associated with sarcopenia risk, suggesting potential benefits of these dietary habits. In contrast, moderate adherence to Dietary Pattern 4 was positively associated with risk. Future interventions should consider personalized dietary thresholds to optimize muscle health in aging populations.

## 1 Introduction

Sarcopenia, marked by the gradual loss of muscle mass, strength, and function, has become a major public health concern amid global aging (1). Beyond the natural aging process, its pathogenesis is influenced by multifactorial interactions, including genetic predispositions, physical inactivity, and nutritional factors (2, 3). This condition greatly elevates the risk of falls, fractures, and functional disability, significantly undermining independence and quality of life in older adults (4). Dietary patterns, which represent the collective composition of food intake, play a crucial role in maintaining musculoskeletal health (5). Optimal diets offer synergistic nutrients crucial for muscle protein synthesis and metabolic balance, potentially slowing sarcopenia progression (6). In contrast, suboptimal diets exacerbate nutritional deficiencies, chronic inflammation, and metabolic dysfunction, thereby accelerating muscle deterioration (7). Although previous studies have underscored the protective effects of individual nutrients, such as protein and vitamin D, a comprehensive dietary approach more effectively captures the interactions between nutrients and reflects real-world eating behaviors (8–10).

PCA has been widely employed to derive data-driven dietary patterns from complex dietary intake datasets, offering more comprehensive insights than single-nutrient analyses (11). Furthermore, RCS regression is beneficial for elucidating potential non-linear associations between dietary patterns score and health outcomes (12). For instance, RCS analyses have revealed U-shaped relationships between a vegetable-staple-fruit dietary pattern score and lipid regulation (13), as well as monotonic positive associations between sodium intake and blood pressure (14). Nonetheless, the non-linear relationships between various dietary patterns score and parameters of sarcopenia, such as physical performance, muscle mass, and grip strength, remain largely unexplored. Traditional linear regression models assume a linear relationship between variables. However, the association between diet and health outcomes might actually be non-linear (15). RCS offers the flexibility to model these non-linear relationships, thereby providing a more accurate and nuanced understanding of how changes in dietary patterns influence the risk of sarcopenia across varying levels of exposure.

This study employs PCA to discern dietary patterns among the elderly and utilizes LR and RCS to thoroughly examine their relationship with sarcopenia. Our aim is to enhance dietary recommendations for preventing and managing sarcopenia in the elderly, thereby improving their muscle health and quality of life.

## 2 Materials and methods

### 2.1 Participants

In Huzhou City, a multi-stage probability-proportional-to-size (PPS) sampling method was implemented. Initially, two streets were selected from each district, and two townships were chosen from each county using PPS sampling. Subsequently, three neighborhood committees and three administrative villages were randomly selected from each street and township, respectively, also employing PPS sampling. Within each of the final sampling units, eligible elderly residents were selected through simple random sampling. The sample size calculation was based on an estimated sarcopenia prevalence of 20%, derived from our pilot study and local literature, with a 95% confidence level, 20% relative precision, a design effect of 2 to accommodate the multi-stage cluster sampling design, and an anticipated response rate of 90%. The minimum required sample size was determined to be 856 participants. Our study successfully enrolled 1,030 community-dwelling elderly individuals, surpassing the calculated minimum requirement and ensuring adequate statistical power for the analysis. The inclusion criteria required that participants be permanent residents at their current address for at least 6 months, possess the physical and cognitive abilities necessary to complete all study procedures, and have the capacity to independently perform the 6-meter walk test. The exclusion criteria included: (1) individuals with physical disabilities or severe chronic conditions that impair mobility (e.g., hemiplegia, diplegia, or severe Chronic Obstructive Pulmonary Disease necessitating long-term oxygen therapy); (2) individuals with implanted medical devices, such as pacemakers, defibrillators, or orthopedic implants containing metal; and (3) participants diagnosed with dementia or severe cognitive impairment, as evidenced by a Mini-Mental State Examination (MMSE) score of less than 18 (16, 17). The Ethical Review Committee of the Huzhou Center for Disease Control and Prevention approved the study protocol, and participants gave written informed consent before participating.

### 2.2 Data collection

Demographic characteristics, lifestyle factors—including physical activity, smoking, and alcohol consumption—and chronic disease history of the participants were collected through face-to-face interviews using a structured questionnaire. The evaluation of chronic disease history was based on a specific inquiry: “Have you ever been diagnosed by a physician with any of the following conditions?” This was followed by a list of conditions, including hypertension, diabetes, dyslipidemia, and cancer. A condition was deemed present if the participant self-reported a physician’s diagnosis. Lifestyle factors were defined as follows: individuals were considered smokers if they smoked at least one cigarette daily for six consecutive months; regular alcohol consumers were those who drank alcohol at least weekly over the past year; and physically active participants met the World Health Organization’s criteria by engaging in ≥150 min of moderate-intensity exercise, ≥75 min of vigorous-intensity exercise, or a combination of both per week (18). Dietary intake was evaluated using a validated food frequency questionnaire (FFQ) certified by the Chinese Center for Disease Control and Prevention. Details of the food items and dietary components in this FFQ are available in prior research (19). Prior to the commencement of the primary investigation, a validation study was conducted on a subsample of 50 participants to evaluate the instrument’s measurement properties. Test–retest reliability was assessed over a six-month period, yielding intra-class correlation coefficients (ICCs) for major food groups that ranged from 0.70 to 0.85, which indicates a high level of reproducibility. In terms of validity, the Pearson correlation coefficients between the FFQ and 3-day 24-h dietary records, used as the reference method, ranged from 0.45 to 0.65 for primary food groups such as meats and vegetables.

### 2.3 Body composition and physical function assessments

This study utilized standardized protocols to evaluate body composition and physical function among all participants. Body composition was evaluated using the Inbody720 device through bioelectrical impedance analysis (BIA). Grip strength was assessed using a calibrated CAMRY dynamometer (model EH 101), while physical performance was gaged via a 6-meter usual gait speed test. The Asian Working Group for Sarcopenia (AWGS) 2019 criteria were used to diagnose sarcopenia (20): low muscle mass was determined by an appendicular skeletal muscle mass index (ASMI) below 7.0 kg/m^2^ for men and 5.7 kg/m^2^ for women; weak grip strength was indicated by handgrip strength under 28 kg for men and 18 kg for women; and slow gait speed was defined as a usual gait speed below 1.0 m/s. Trained professionals collected fasting venous blood samples for high-sensitivity C-reactive protein (hs-CRP) analysis. All assessments were conducted in a controlled environment maintained at a temperature of 22–24 °C, with daily calibration of equipment. The assessments were performed by professionally trained technicians, ensuring high inter-rater reliability (ICC > 0.90), and strict adherence to standard operating procedures was observed.

### 2.4 Statistical methods

Statistical analyses were conducted using SPSS 25.0 (IBM) and R 4.2.3. Continuous variables were described as mean and standard deviation for normally distributed data, analyzed with t-tests, or as median and interquartile range for non-normally distributed data, assessed using Mann–Whitney U tests. Categorical variables were described as frequencies and percentages, and analyzed using chi-square tests. Dietary patterns were identified through PCA with varimax rotation applied to food frequency data, and were subsequently named based on the predominant food groups. Adherence scores for dietary patterns were determined by summing the weighted food group intakes, and participants were divided into tertiles (T1-T3) based on factor score distribution. LR was employed to examine the associations between adherence to dietary patterns, as categorized by tertiles, and the presence of sarcopenia. Nonlinear relationships were evaluated using RCS with knots at the 5th, 35th, 65th, and 95th percentiles of factor scores, using the 5th percentile as the reference. All RCS analyses, including model fitting and curve plotting, were performed using the rms and plotRCS packages in R version 4.2.3. A *p*-value below 0.05 was deemed statistically significant.

## 3 Results

### 3.1 General demographic characteristics of participants

In this study, a cohort of 1,030 community-dwelling older adults was examined. The overall prevalence of sarcopenia within the sample was determined to be 21.2% (*n* = 218). Table 1 delineates the baseline characteristics of the participants, categorized according to sarcopenia status. It was observed that participants with sarcopenia were significantly older than those without the condition (74.2 ± 6.4 years vs. 69.6 ± 5.9 years, *p* < 0.01). No significant differences were observed between the two groups in terms of gender distribution (*p* = 0.947), educational level (*p* = 0.102), smoking status (*p* = 0.206), or the prevalence of diabetes (*p* = 0.087), dyslipidemia (*p* = 0.370), and cancer (*p* = 0.245). However, several factors were significantly associated with sarcopenia status. A lower proportion of individuals with sarcopenia reported current alcohol consumption (17.0% vs. 23.4%, *p* < 0.05) and engagement in physical activity (43.6% vs. 55.0%, *p* < 0.01). Additionally, a significant difference was identified in the prevalence of hypertension, with a lower proportion of hypertensive individuals present in the sarcopenia group (50.9% vs. 60.7%, *p* < 0.01). As anticipated, participants with sarcopenia exhibited a significantly lower body mass index (BMI) (21.17 ± 3.35 kg/m^2^ vs. 24.29 ± 3.98 kg/m^2^, *p* < 0.01). A small yet statistically significant difference was noted in sleep duration, with the sarcopenia group reporting longer sleep hours (8.31 ± 1.57 vs. 7.84 ± 1.39, *p* < 0.01). Participants with sarcopenia exhibited a significantly lower total energy intake (1856.4 ± 756.6 kcal vs. 2234.4 ± 701.4 kcal, *p* < 0.01). No significant difference was observed in high-sensitivity C-reactive protein (hs-CRP) levels between the groups.

**Table 1:** Demographic and clinical characteristics of study participants stratified by sarcopenia status (*N* = 1,030).

Variable	Sarcopenia		All (*n* = 1,030)	*p*
	No (*n* = 812)	Yes (*n* = 218)		
Gender, *n* (%)				
Male	382 (47.0)	102 (46.8)	484 (47.0)	0.947
Female	430 (53.0)	116 (53.2)	546 (53.0)	
Age, years [mean (SD)]	69.6 (5.9)	74.2 (6.4)	70.5 (6.3)	<0.01*
Education level, *n* (%)				
Primary or below	560 (69.0)	164 (75.2)	724 (70.3)	0.102
Secondary	201 (24.8)	47 (21.6)	248 (24.1)	
Tertiary or above	51 (6.3)	7 (3.2)	58 (5.6)	
Smoking status, *n* (%)				
No	663 (81.7)	186 (85.3)	849 (82.4)	0.206
Yes	149 (18.3)	32 (14.7)	181 (17.6)	
Alcohol consumption, *n* (%)				
No	622 (76.6)	181 (83.0)	803 (78.0)	<0.05*
Yes	190 (23.4)	37 (17.0)	227 (22.0)	
Physical activity, *n* (%)				
No	365 (45.0)	123 (56.4)	488 (47.4)	<0.01*
Yes	447 (55.0)	95 (43.6)	542 (52.6)	
Hypertension, *n* (%)				
No	319 (39.3)	107 (49.1)	426 (41.4)	<0.01*
Yes	493 (60.7)	111 (50.9)	604 (58.6)	
Diabetes, *n* (%)				
No	635 (78.2)	182 (83.5)	817 (79.3)	0.087
Yes	177 (21.8)	36 (16.5)	213 (20.7)	
Dyslipidemia, *n* (%)				
No	701 (86.3)	183 (83.9)	884 (85.8)	0.370
Yes	111 (13.7)	35 (16.1)	146 (14.2)	
Cancer, *n* (%)				
No	802 (98.8)	213 (97.7)	1,015 (98.5)	0.245
Yes	10 (1.2)	5 (2.3)	15 (1.5)	
BMI, (kg/m^2^) [mean (SD)]	24.3 (4.0)	21.2 (3.4)	23.6 (3.3)	<0.01*
Sleep duration, hour [mean (SD)]	7.8 (1.4)	8.3 (1.6)	7.9 (1.4)	<0.01*
hs-CRP, (mg/L) [M(IQR)]	1.9 (1.5)	2.1 (1.9)	2.0 (1.6)	0.083
Total energy,(kcal)	2234.4 (701.4)	1856.4 (756.6)	2154.4 (733.1)	<0.01*

Age, Body mass index (BMI) Sleep duration and Total energy are expressed as mean (SD), using *t*-tests; hs-CRP is expressed as the median (M) and interquartile range (IQR), which is used for data with non-normal distributions using Mann–Whitney U tests to compare. *p < 0.05.

### 3.2 Dietary patterns characteristic

A principal component factor analysis (KMO = 0.84; Bartlett’s test of sphericity, *p* < 0.001) revealed four dietary patterns, explaining 53.82% of the variance. These patterns were characterized by higher factor loadings: Dietary Pattern 1 (plant-based whole grain-legume pattern) explained 20.51% of the variance; Dietary Pattern 2 (traditional high meat-egg pattern) accounted for 13.37%; Dietary pattern 3 (vegetable-freshwater aquatic pattern) contributed 12.04%; and Dietary Pattern 4 (high dairy-low refined grain pattern) represented 7.91%. Detailed factor loadings and variance contributions are provided in Tables 2, 3.

**Table 2:** Characterization of four dietary patterns based on food group loadings.

Food type	Dietary pattern†			
	Pattern 1	Pattern 2	Pattern 3	Pattern 4
Tubers	0.772			
Soybean and products	0.743			
Mushrooms and fungi	0.717			
Legumes	0.710			
Nuts	0.469			
Wheat	0.383			
Egg		0.762		
Red meat		0.668	0.374	
Poultry		0.654		
Fruits		0.383		
Vegetable			0.750	
Freshwater aquatic			0.649	
Milk				0.699
Rice			0.449	−0.605

†The dietary patterns were interpreted and named based on high factor loadings (≥0.35).

**Table 3:** Cumulative variance contribution rates of four patterns.

Principal component	Sum	Variance (%)	Cumulative variance contribution rate (%)
Pattern 1	2.87	20.51	20.51
Pattern 2	1.87	13.37	33.87
Pattern 3	1.69	12.04	45.91
Pattern 4	1.11	7.91	53.82

### 3.3 Associations between dietary patterns scores and the risk of sarcopenia

Table 4 presents the results of logistic regression analyses investigating the associations between four dietary patterns and the risk of sarcopenia across progressively adjusted models. Model 1 was the crude, unadjusted model. Model 2 was adjusted for gender, age, and education level. Model 3 was further adjusted for smoking status, alcohol consumption, physical activity, hypertension, diabetes, dyslipidemia, cancer, BMI, sleep duration, and hs-CRP. (1) Dietary Pattern 1 did not exhibit a statistically significant association with sarcopenia across any of the models examined (Model 3, *p* = 0.30). Although the OR for the third tertile (T3) was below 1, it did not achieve statistical significance when compared to the reference group (T1) in the fully adjusted model (aOR = 0.77, 95%CI: 0.48–1.23, *p* = 0.28). (2) Dietary Pattern 2 demonstrated a significant inverse association with sarcopenia across all models (Model 3, *p* < 0.05). In the fully adjusted Model 3, individuals in T3 exhibited a significantly reduced risk of sarcopenia compared to those in T1 (aOR = 0.63, 95% CI: 0.40–0.98, *p* < 0.05). (3) Dietary Pattern 3 exhibited the most pronounced inverse relationship with the risk of sarcopenia, as demonstrated in Model 3 (*p* < 0.01). The likelihood of sarcopenia diminished progressively with an increase in the pattern score, transitioning from T2 to T3. Specifically, in Model 3, the aOR for T2 was 0.63 (95% CI: 0.40–0.97); this protective effect was further amplified in T3, where the aOR was 0.48 (95% CI: 0.29–0.78, *p* < 0.01). (4) Dietary Pattern 4 was positively associated with an increased risk of sarcopenia. In Model 3, the second tertile (T2) was associated with a significantly elevated risk (aOR = 1.73, 95% CI: 1.10–2.72, *p* < 0.05), although the association for T3 did not reach statistical significance (aOR = 1.29, 95% CI: 0.80–2.08, *p* = 0.20). BMI inherently reflects long-term energy balance and serves as an effective control for energy-related confounding variables in body composition analyses. Adjusting for total energy intake had minimal impact on the direction, magnitude, and statistical significance of the associations between dietary patterns and sarcopenia. The consistency of results across different models (Model 3, adjusted for energy intake in Model 4, and adjusted for both energy intake and BMI in Model 5) highlights the robustness of our findings (Appendix Table S1). Adjustments for total energy intake, whether alone or in conjunction with BMI, did not significantly alter the key associations for Patterns 3 and 4, which remained statistically significant and consistent in direction. Although there was a slight attenuation in the association of Pattern 2 in energy-adjusted models, this does not affect the primary conclusions. These findings confirm that the fundamental relationships between dietary patterns and sarcopenia are resilient to adjustments for energy intake.

**Table 4:** Logistic regression analysis of dietary patterns and sarcopenia in elderly adults.

Dietary pattern	Factor score tertiles	Model 1		Model 2		Model 3	
		*OR* (95 *CI*%)	*p*	*aOR* (95 *CI*%)	*p*	*aOR* (95 *CI*%)	*p*
Pattern 1			0.05		0.08		0.30
	T1	1		1		1	
	T2	1.10 (0.77–1.57)	0.59	1.14 (0.78–1.65)	0.50	1.11 (0.72–1.69)	0.64
	T3	0.70 (0.48–1.02)	0.07	0.73 (0.49–1.08)	0.12	0.77 (0.48–1.23)	0.28
Pattern 2			<0.01		<0.01		<0.05^*^
	T1	1		1		1	
	T2	0.92 (0.65–1.30)	0.63	0.95 (0.66–1.37)	0.77	1.01(0.67–1.52)	0.98
	T3	0.47 (0.32–0.70)	<0.01	0.57 (0.38–0.85)	<0.01	0.63 (0.40–0.98)	<0.05^*^
Pattern 3			<0.01		<0.01		<0.01^*^
	T1	1		1		1	
	T2	0.51 (0.36–0.73)	<0.01	0.51 (0.35–0.73)	<0.01	0.63 (0.40–0.97)	<0.05^*^
	T3	0.28 (0.19–0.41)	<0.01	0.33 (0.22–0.50)	<0.01	0.48 (0.29–0.78)	<0.01^*^
Pattern 4			<0.01		<0.05		<0.05^*^
	T1	1		1		1	
	T2	2.07 (1.42–3.03)	<0.01	1.89 (1.27–2.81)	<0.01	1.73 (1.10–2.72)	<0.05^*^
	T3	1.51 (1.02–2.24)	<0.05	1.47 (0.96–2.24)	0.08	1.29 (0.80–2.08)	0.20

Model 1: Crude model. Model 2 adjusted by gender, age, and education level. Model 3 adjusted by gender, age, education level, smoking status, alcohol consumption, physical activity, hypertension, diabetes, dyslipidemia, cancer, BMI, sleep duration, and hs-CRP. ^*^p < 0.05.

**Figure 1:**
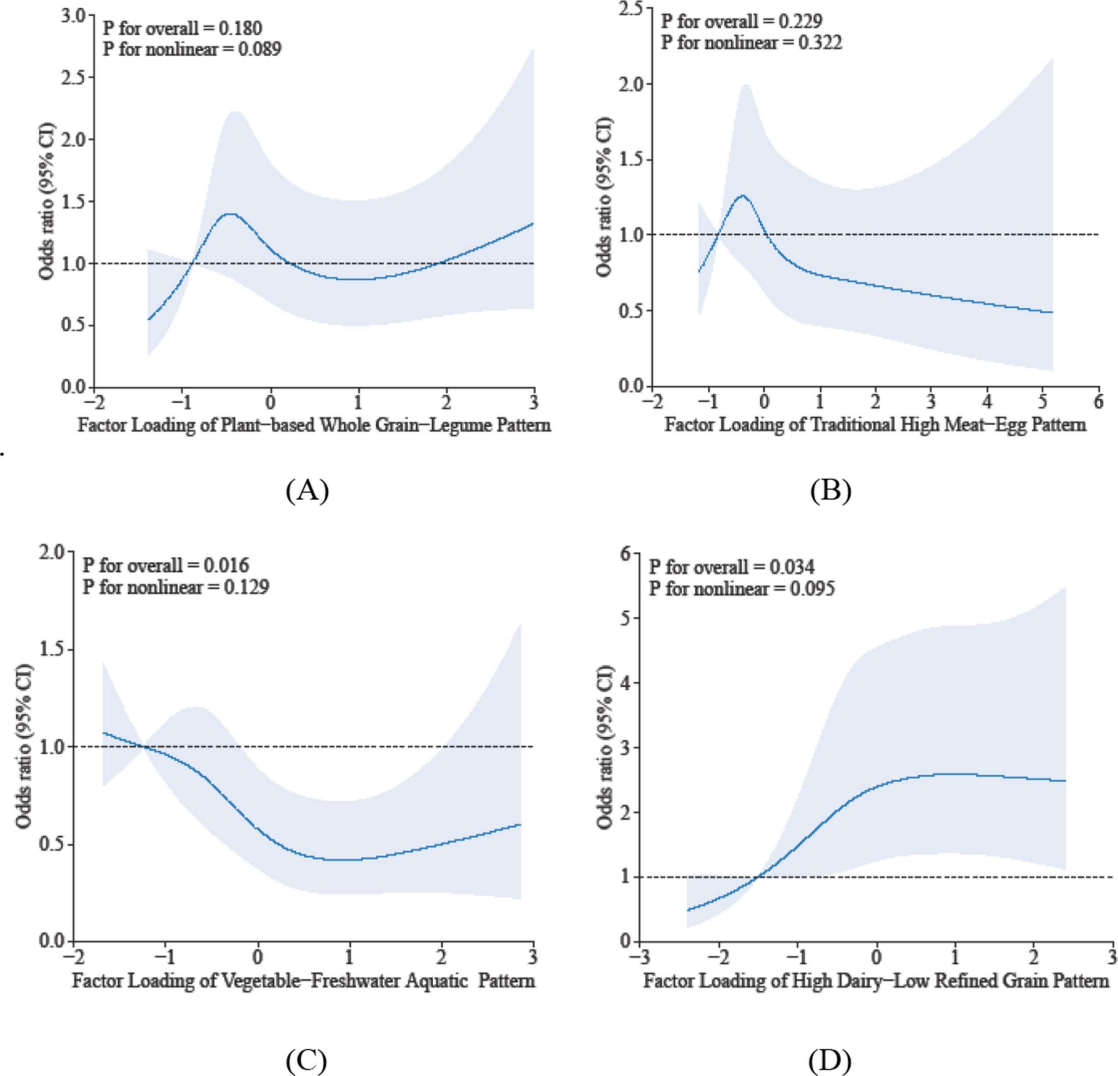
Dose-reponse associations between different dietary patterns **(A)** Dietary Pattern 1, **(B)** Dietary Pattern 2, **(C)** Dietary Pattern 3, **(D)** Dietary Pattern 4 and sarcopenia. All models were adjusted for gender, age, education level, smoking status, alcohol consumption, physical activity, hypertension, diabetes, dyslipidemia, cancer, BMI, sleep duration, and hs-CRP.

Restricted cubic spline analyses identified distinct dose–response associations between dietary patterns and sarcopenia risk: (1) Dietary Pattern 1 did not demonstrate a significant linear association with sarcopenia risk (P-overall = 0.180), the RCS analysis indicated a non-linear pattern of borderline significance (P-nonlinearity = 0.089). The observed relationship was characterized by a U-shaped trend, wherein both the lowest and highest factor scores were associated with a non-significant increase in risk compared to intermediate scores. (2) For Dietary Pattern 2, the inflection point suggests that below factor scores of −0.75, the risk of sarcopenia increases with higher pattern scores, while above −0.75, the risk decreases. However, since the overall association (*p* = 0.229) and nonlinearity (*p* = 0.322) were not statistically significant, this finding should be viewed cautiously as exploratory. It highlights a potential transition in risk, but further studies with larger samples are needed for confirmation. (3) Dietary Pattern 3 showed a significant inverse association overall (P-overall <0.05), with a nonlinear trend where risk initially decreased with greater adherence but experienced a modest rebound at extreme intake levels (*p*-nonlinearity >0.05). (4) Dietary Pattern 4 exhibited a statistically significant positive association with the risk of sarcopenia (*p*-overall < 0.05). Furthermore, there was evidence of marginally significant nonlinearity (*p*-nonlinearity = 0.095). This nonlinear trend is characterized by a marked increase in the risk of sarcopenia at moderate levels of adherence to the pattern, which subsequently stabilizes at higher adherence levels. At moderate adherence, the risk of sarcopenia increases significantly, potentially due to an “imbalanced interaction” between dairy and refined grains: even a high intake of dairy is insufficient to offset the muscle-supporting deficiency caused by low consumption of refined grains. Collectively, these findings suggest that suboptimal adherence—whether insufficient or excessive—to specific dietary patterns may increase health risks. The observed nonlinear dose–response relationships indicate potential intake thresholds that warrant further investigation. The results are shown in Figure 1.

### 3.4 Dose–response associations of dietary pattern scores with muscle mass, physical performance and grip strength

**Figure 2:**
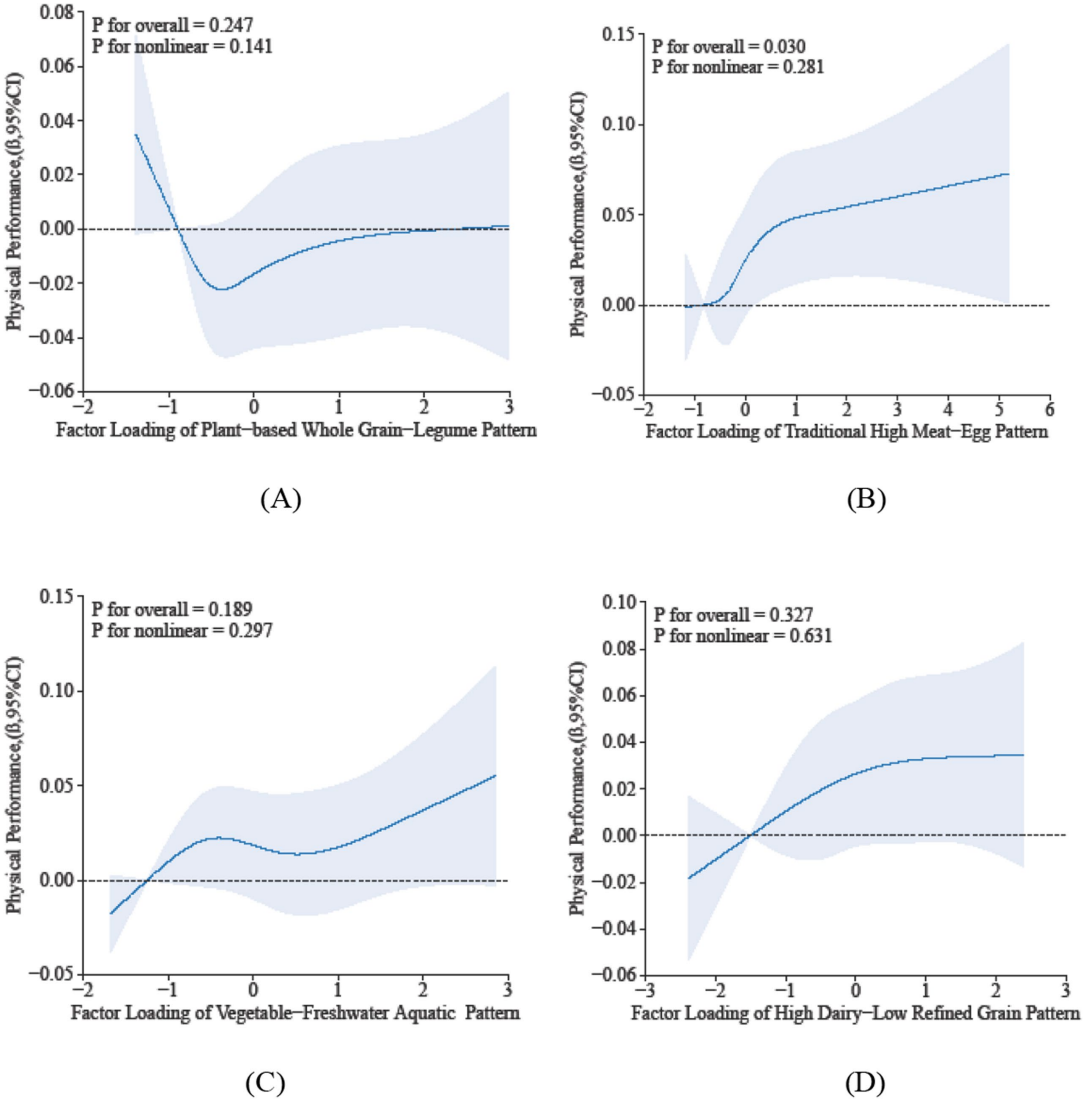
Dose-reponse associations between different dietary patterns **(A)** Dietary Pattern 1, **(B)** Dietary Pattern 2, **(C)** Dietary Pattern 3, **(D)** Dietary Pattern 4 and physical performance. All models were adjusted for gender, age, education level, smoking status, alcohol consumption, physical activity, hypertension, diabetes, dyslipidemia, cancer, BMI, sleep duration, and hs-CRP.

**Figure 3:**
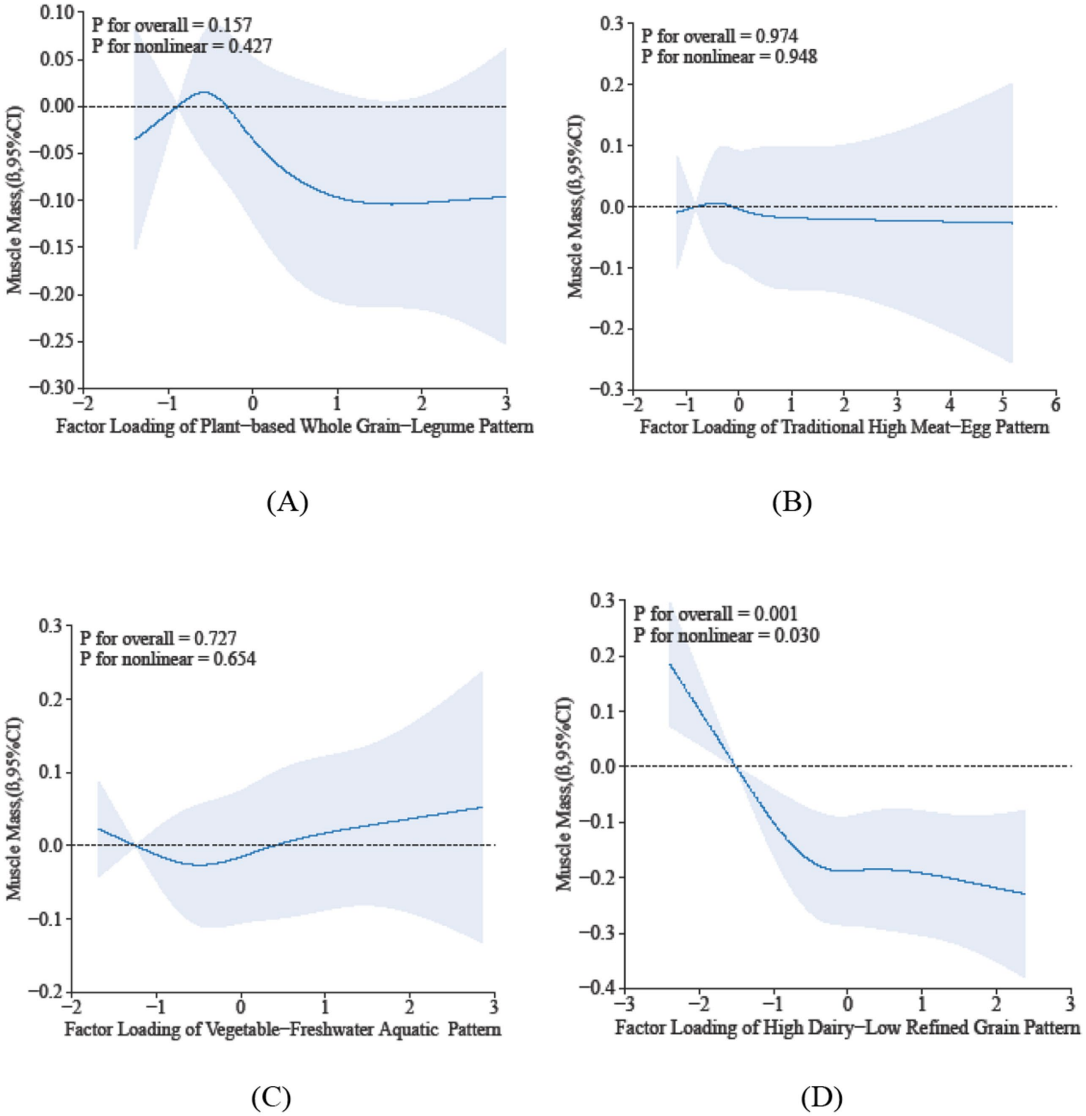
Dose-reponse associations between different dietary patterns **(A)** Dietary Pattern 1, **(B)** Dietary Pattern 2, **(C)** Dietary Pattern 3, **(D)** Dietary Pattern 4 and muscle mass. All models were adjusted for gender, age, education level, smoking status, alcohol consumption, physical activity, hypertension, diabetes, dyslipidemia, cancer, BMI, sleep duration, and hs-CRP.

**Figure 4:**
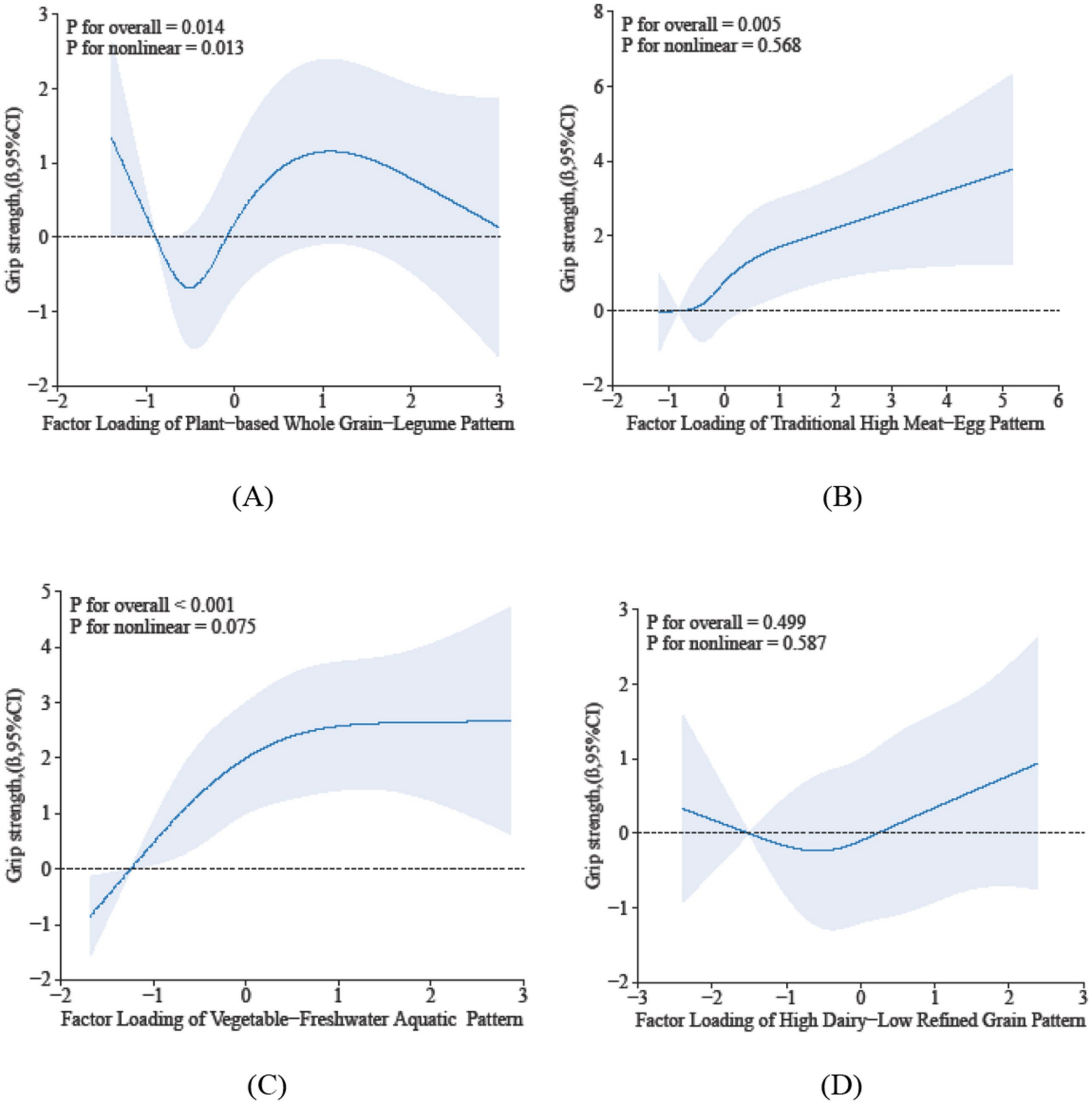
Dose-reponse associations between different dietary patterns **(A)** Dietary Pattern 1, **(B)** Dietary Pattern 2, **(C)** Dietary Pattern 3, **(D)** Dietary Pattern 4 and grip strength. All models were adjusted for gender, age, education level, smoking status, alcohol consumption, physical activity, hypertension, diabetes, dyslipidemia, cancer, BMI, sleep duration, and hs-CRP.

The restricted cubic spline analysis elucidates the dose–response relationship between sarcopenia parameters and dietary pattern scores. Specifically, Dietary Pattern 2 demonstrated a significant positive association with physical performance (P-overall < 0.05), characterized by a monotonically increasing trend that plateaued at higher scores, despite the absence of significant nonlinearity (*p* = 0.281) in Figure 2. In contrast, Dietary Pattern 4 was inversely associated with muscle mass in a nonlinear manner (*p*-overall < 0.05, *p*-nonlinearity < 0.05) in Figure 3. Notably, both Dietary Pattern 2 and Dietary Pattern 3 exhibited similar linear positive associations with grip strength (*p*-overall < 0.05 for both), with no evidence of nonlinearity (*p* > 0.05) in Figure 4, indicating proportional benefits across varying intake levels for these two distinct dietary approaches.

## 4 Discussion

The prevalence of sarcopenia has significantly risen in recent years. In our cross-sectional study, we identified 218 cases of sarcopenia, representing 21.2% of the 1,030 participants. These findings align with a recent meta-analysis indicating a prevalence of 10–27% among global populations aged 60 years and older, which correlates with economic development and the accompanying lifestyle and dietary changes (1, 21). As global aging trends continue to accelerate, the identification of modifiable risk factors, particularly those related to dietary habits, has become essential for the prevention and management of sarcopenia (22). However, existing research on the relationship between diet and sarcopenia has predominantly concentrated on individual nutrients or food groups, resulting in a gap in our understanding of how comprehensive dietary patterns affect muscle health in older adults (8, 9). Huzhou City, situated in Zhejiang Province in eastern China and adjacent to Taihu Lake—one of the country’s four principal freshwater lakes—provides a distinctive setting for examining these relationships (23). The area’s unique culinary traditions, which emphasize freshwater aquatic products, rice-based staples, and soy-derived foods, present specific advantages for conducting dietary studies (24).

The prevalence of sarcopenia shows a significant age-related increase, likely mediated by the decline in anabolic hormones such as testosterone and growth hormone, which are crucial for muscle maintenance (25). Physically active participants demonstrated a markedly lower prevalence of sarcopenia, thereby corroborating the extensively documented protective role of regular exercise in mitigating muscle loss among older adults. This observation aligns with a substantial body of literature indicating that physical activity enhances muscle protein synthesis and attenuates age-related sarcopenia (26, 27). The elevated prevalence of sarcopenia observed in the non-drinking cohort, as opposed to the drinking cohort, may be ascribed to the potential anti-inflammatory properties of alcohol, which could mitigate sarcopenia risk by reducing inflammatory markers (28). The significant differences noted in both BMI (decreased) and sleep duration (increased) within the sarcopenia group suggest complex interactions between body composition regulation, sleep patterns, and muscle metabolism in aging populations (27). Our univariate analysis showed a lower prevalence of hypertension in the sarcopenia group, which seemed paradoxical. However, after adjusting for age and BMI in a multivariate analysis, this link was not statistically significant (OR = 0.83, 95% CI: 0.580–1.199, *p* = 0.326). This indicates that the initial difference was likely due to confounding factors, particularly the strong positive link between age and sarcopenia and the negative link with BMI. Although our study did not reveal statistically significant differences in sarcopenia prevalence based on gender, smoking status, educational attainment, or hs-CRP, these associations merit further exploration in subsequent research. Furthermore, our findings identified specific dietary patterns that are significantly associated with sarcopenia risk.

### 4.1 Relationship of dietary pattern 2 (traditional high meat-egg pattern) with sarcopenia risk

The findings of this study elucidate a complex relationship between Dietary Pattern 2 and the risk of sarcopenia among the elderly. Dietary Pattern 2, characterized by a high consumption of eggs, red meat, and poultry, was found to have an inverse association with the prevalence of sarcopenia in logistic regression analysis. However, given the observational nature of this study, this association should not be construed as causal. It is plausible that individuals with superior overall health and muscle status are more inclined to consume such animal-based protein sources. Consequently, while these findings are consistent with previous research suggesting that high-quality protein supports muscle protein synthesis and may mitigate age-related muscle loss (29), further longitudinal or interventional studies are necessary to elucidate any potential causal protective effects of animal-based proteins against sarcopenia. The RCS analysis indicated a U-shaped trend, although not statistically significant, between the dietary pattern score and sarcopenia risk. This non-linear pattern may imply that both insufficient and excessive intake of this dietary pattern could affect muscle health, with a potential inflection point where moderate consumption confers the lowest risk. Significant linear dose–response relationships were observed between the dietary pattern score and both physical performance and grip strength, suggesting that increased adherence to this animal protein-rich pattern may progressively enhance muscle function. This finding aligns with existing evidence that dietary protein, particularly when combined with physical activity, enhances muscle strength and physical capacity in older adults (30). In summary, although Dietary Pattern 2 exhibits inverse associations with sarcopenia, potentially indicating a U-shaped relationship that underscores the importance of moderate consumption, its robust linear correlations with muscle function indicators are particularly significant. Nevertheless, these conclusions are based on cross-sectional analyses and should be approached with caution. To substantiate the potential implications of this dietary pattern for geriatric nutrition strategies, further validation through longitudinal or interventional studies is necessary.

### 4.2 Relationship of dietary pattern 3 (vegetable-freshwater aquatic pattern) with sarcopenia risk

The present study identified a negative association between dietary pattern 3—characterized by high consumption of vegetables, freshwater aquatic foods, and rice—and the risk of sarcopenia (i.e., higher adherence to this dietary pattern score was associated with a reduced odds of sarcopenia), as determined through logistic regression analysis. This inverse relationship is consistent with existing evidence suggesting that plant-based diets rich in antioxidants, along with aquatic foods high in quality protein, may synergistically mitigate muscle loss in aging populations (31). Further analysis using RCS revealed a significant linear dose–response relationship between the factor score of this dietary pattern and sarcopenia risk, indicating that each unit increase in adherence provides incremental benefits. However, the observed plateau and subsequent downward trend at higher factor scores, although not statistically significant, may suggest a potential ceiling effect, where maximal protective benefits are achieved at moderate-to-high intake levels. A significant linear dose–response relationship was identified between the dietary pattern score and grip strength, thereby reinforcing the biological plausibility of this pattern’s role in maintaining muscle function. This observation supports the findings of intervention studies that indicate consuming fish twice weekly for 10 weeks can improve sarcopenia parameters, muscle mass, and function in middle-aged and older adults (32). In conclusion, dietary pattern 3 exhibits clinically meaningful, dose-dependent associations with the prevention of sarcopenia. Its traditional composition renders it particularly pertinent for public health strategies targeting aging populations in Asia.

### 4.3 Relationship of dietary pattern 4 (high dairy-low refined grain pattern) with sarcopenia risk

The present study elucidated complex relationships between dietary pattern 4—characterized by a positive loading for dairy and a negative loading for rice—and the risk of sarcopenia. Logistic regression analysis indicated a significant positive association in the second tertile (T2) of factor scores (OR = 1.88, 95% CI 1.20–2.95); however, this association diminished in the highest tertile (T3) (OR = 1.36, 95% CI 0.85–2.18). These findings suggest a threshold effect, wherein moderate adherence to this dietary pattern is associated with an increased risk of sarcopenia. Furthermore, at extreme levels of intake, adaptive metabolic responses may mitigate the initial adverse effects. A previous study suggested that a monotonous dietary pattern was positively associated with the risk of sarcopenia, but this research did not account for potential threshold effects (19). Possible explanations encompass the diminished glucose utilization in aging muscle, attributed to a reduction in complex carbohydrates derived from rice. Concurrently, the potential for excessive dairy consumption to ultimately provide adequate nutrients that mitigate the initial risks is considered. Prior research has indicated that dairy proteins can sustain muscle protein synthesis by enhancing amino acid availability, even under conditions of low glycogen (33). The RCS analysis elucidates several critical aspects. A significant positive linear dose–response relationship is identified between factor scores and sarcopenia risk (*p*-overall association < 0.05; *p*-non-linear association > 0.05), which supports the findings from the logistic regression analysis. A plateau effect is noted at higher scores, consistent with the attenuation observed in the tertile-based analysis. Furthermore, a significant nonlinear relationship with muscle mass is detected (*p*-overall association < 0.05; *p*-non-linear association < 0.05), indicating potential inflection points in its impact on body composition. In summary, Dietary Pattern 4 exhibits a complex association with sarcopenia, with risk associations being most pronounced among individuals with moderate adherence. Consequently, individuals with moderate adherence to this dietary pattern are likely to experience the most substantial benefits from intervention strategies.

Our study examines the impact of dietary transitions on the development of sarcopenia among community-dwelling older adults in eastern China. This study possesses several significant strengths, notably the utilization of a substantial community-based sample (*n* = 1,030) and the implementation of the standardized AWGS 2019 criteria for diagnosing sarcopenia, both of which contribute to the robustness of the findings. From a methodological perspective, the integration of principal component analysis with restricted cubic spline models facilitated the identification of culturally specific dietary patterns and elucidated dose–response relationships between these patterns and the risk of sarcopenia. This approach advances associative evidence to a more sophisticated level. The insights derived from this study provide a valuable foundation for the development of targeted nutritional strategies tailored to specific populations. Our analysis reveals distinct relationships between dietary patterns and sarcopenia risk, with potential practicality implications. Dietary Pattern 2 displayed a protective effect; specifically, individuals in the highest adherence group (T3) experienced a 37% reduction in the risk of sarcopenia compared to those in the lowest adherence group (T1). The protective effect of Dietary Pattern 3 was the most pronounced, the risk of sarcopenia was reduced by 37% in the moderate adherence group (T2) and further reduced by 52% in the highest adherence group (T3). Conversely, Dietary Pattern 4 demonstrated a detrimental threshold effect: compared to the T1 group, the risk of sarcopenia increased significantly by 73% in the moderate adherence group (T2), indicating that the peak risk occurs at this level. These findings underscore the importance of achieving the highest adherence to protective dietary patterns (Patterns 2 and 3) to maximize health benefits, while even moderate adherence to monotonous dietary patterns (Pattern 4) leads to a substantial increase in risk (73%) and should be avoided. In relation to RCS inflection points, our analyses did not identify statistically significant inflection points. This outcome may be attributed to the inherent characteristics of posteriori dietary patterns derived through PCA, as opposed to *a priori* patterns such as the Mediterranean diet scores, which possess established and consistent structures and for which RCS methods are more frequently validated (34). Consequently, tertile scores seem more appropriate for delineating thresholds in posteriori-derived dietary patterns, as they correspond with the exploratory objective of identifying novel dietary structures and their graded associations with health outcomes. Future research focusing on a priori dietary patterns might benefit from employing RCS to refine continuous thresholds. The findings of this study should be considered with an awareness of certain limitations. Initially, the cross-sectional design imposes two primary limitations: it precludes the establishment of causal inferences and fails to completely eliminate the potential influence of reverse causality. This is exemplified in the scenario where early subclinical sarcopenia could lead to alterations in dietary preferences, rather than dietary patterns directly impacting the risk of sarcopenia. Furthermore, the study’s concentration on an Asian population from a specific geographic region, alongside the exclusion of individuals with severe chronic conditions, may limit the generalizability of the findings. Additionally, measurement error inherent in dietary assessment may have attenuated the observed associations toward the null. Finally, unmeasured endocrine factors are a major limitation, as these hormones regulate muscle synthesis and degradation, affecting sarcopenia risk directly and indirectly. The absence of data on these hormones introduces unquantified bias. Additionally, the lack of endocrine biomarker data and the emphasis on food groups instead of specific nutrients limit mechanistic insights. Future studies should include detailed energy intake, endocrine biomarker data, and precise dietary information to validate findings and clarify biological mechanisms.

## 5 Conclusion

This research elucidates the intricate and diverse relationships between specific dietary patterns and the risk of sarcopenia among the elderly population. The vegetable-freshwater aquatic dietary pattern and traditional high meat-egg pattern are both associated with a reduced risk of sarcopenia, highlighting the necessity of maintaining a balanced intake to achieve optimal health benefits. In contrast, the high dairy-low refined grain pattern may demonstrate a threshold effect, wherein moderate adherence could potentially increase the risk of sarcopenia. These findings emphasize the critical importance of precision nutrition strategies that take into account both the type of dietary pattern and the optimal intake levels.

## Data Availability

The datasets generated and analyzed during the current study are available from the corresponding authors upon reasonable request. Requests to access the datasets should be directed to 614094266@qq.com.
