# Supplementary material for: Associations between dietary patterns and sarcopenia in aging populations: a community study from eastern China’s Huzhou city
Source: Front Nutr. 2025 Nov 25;12:1677335. doi: 10.3389/fnut.2025.1677335 (PMC12685659; doi:10.3389/fnut.2025.1677335)
Supplement: Supplementary file 1 [file Table_1.docx]

**Appendix**

**Table S1 Logistic regression analysis of dietary patterns and sarcopenia in elderly adults**

| Dietary Pattern |  | Model 4 | | Model 5 | |
| --- | --- | --- | --- | --- | --- |
|  |  | *aOR*(95 *CI*%) | *p* | *aOR*(95 *CI*%) | *p* |
| Pattern 1 |  |  | 0.45 |  | 0.68 |
|  | T1 | 1 |  | 1 |  |
|  | T2 | 1.29(0.87-1.89) | 0.21 | 1.17(0.76-1.80) | 0.47 |
|  | T3 | 1.20(0.71-.2.03) | 50 | 0.98(0.55-1.74) | 0.94 |
| Pattern 2 |  |  | 0.24 |  | 0.22 |
|  | T1 | 1 |  | 1 |  |
|  | T2 | 0.97(0.67-1.42) | 0.88 | 1.06(0.70-1.62) | 0.77 |
|  | T3 | 0.70(0.45-1.09) | 0.12 | 0.71(0.43-1.15) | 0.16 |
| Pattern 3 |  |  | <0.01^*^ |  | <0.05^*^ |
|  | T1 | 1 |  | 1 |  |
|  | T2 | 0.61(0.41-0.92) | <0.05 | 0.63(0.40-0.99) | <0.05^*^ |
|  | T3 | 0.42(0.26-0.69) | <0.01 | 0.50(0.29-0.87) | <0.05^*^ |
| Pattern 4 |  |  | <0.05^*^ |  | <0.05^*^ |
|  | T1 | 1 |  | 1 |  |
|  | T2 | 1.60(1.04-2.46) | <0.05 | 1.62(1.03-2.55) | <0.05^*^ |
|  | T3 | 1.44(0.93-2.21) | 0.10 | 1.34(0.85-2.11) | 0.21 |

Note: Model 4 adjusted by gender, age, education level, smoking status, alcohol consumption, physical activity, hypertension, diabetes, dyslipidemia, cancer, sleep duration, hs-CRP, energy intake. Model 5 adjusted by gender, age, education level, smoking status, alcohol consumption, physical activity, hypertension, diabetes, dyslipidemia, cancer, sleep duration, hs-CRP, both energy intake and BMI.* p < 0.05.
